# Outpatient treatment costs and their potential impact on cancer care

**DOI:** 10.1002/cam4.308

**Published:** 2014-07-24

**Authors:** Takahiro Isshiki

**Affiliations:** Department of Hematology, Teikyo University Chiba Medical Center3426-3 Anesaki, Ichihara, 299-0111, Chiba, Japan

**Keywords:** Cancer, cost, electronic medical record data, outpatient treatment, time

## Abstract

Cancer creates a tremendous financial burden. Cancer-related costs are categorized into direct, indirect, and psychosocial costs. Although there have been many reports on medical care costs, which are direct, those on other costs are extremely scarce. We estimated travel time and costs required for cancer patients to receive outpatient treatment. We studied 521 cancer patients receiving anti-cancer treatment between February 2009 and December 2012 at the Outpatient Chemotherapy Center of Teikyo University Chiba Medical Center. Address data were extracted from Data Warehouse electronic medical records, and travel distance and time required for outpatient treatment were calculated via MapInfo and ACT Distance Calculator Package. Transportation costs were estimated on the basis of ¥274 (=$3.00) per kilometer. The study design was approved by an ethics review board of Teikyo University (12–851). Average round-trip travel distance, time, and cost for all patients were 26.7 km, 72.5 min, and ¥7,303 ($79.99), respectively. Cancer patients incurred a travel cost of ¥4000–¥9000 ($40.00 to $100.00) for each outpatient treatment. With population aging, seniors living alone and senior households are increasing, and outpatient visits are becoming a common burden.

## Introduction

Cancer creates a tremendous financial burden [1]. Cancer-related costs are categorized into direct, indirect, and psychosocial costs. Although many reports are available on medical care costs, which are direct, those on other costs are extremely scarce [2]. We estimated travel time and costs required for cancer patients to receive outpatient treatment.

## Methods

We studied 521 cancer patients receiving anti-cancer treatment between February 2009 and December 2012 at the Outpatient Chemotherapy Center of Teikyo University Chiba Medical Center. Address data were extracted from Data Warehouse electronic medical records. Travel distance and time required for outpatient treatment were calculated via software MapInfo® (Pitney Bowes Software K.K., Tokyo, Japan) and ACT Distance Calculator Package® (Advanced Core Technologies, Inc., Tokyo, Japan). Transportation costs were estimated on the basis of ¥274 (=$3.00) per kilometer (http://www.civil.eng.osaka-u.ac.jp/plan/staff/inoi/keikaku08_3.pdf). The study design was approved by an ethics review board of Teikyo University (12–851). Address data were anonymized and de-identified prior to analysis.

## Results

**Figure 1:**
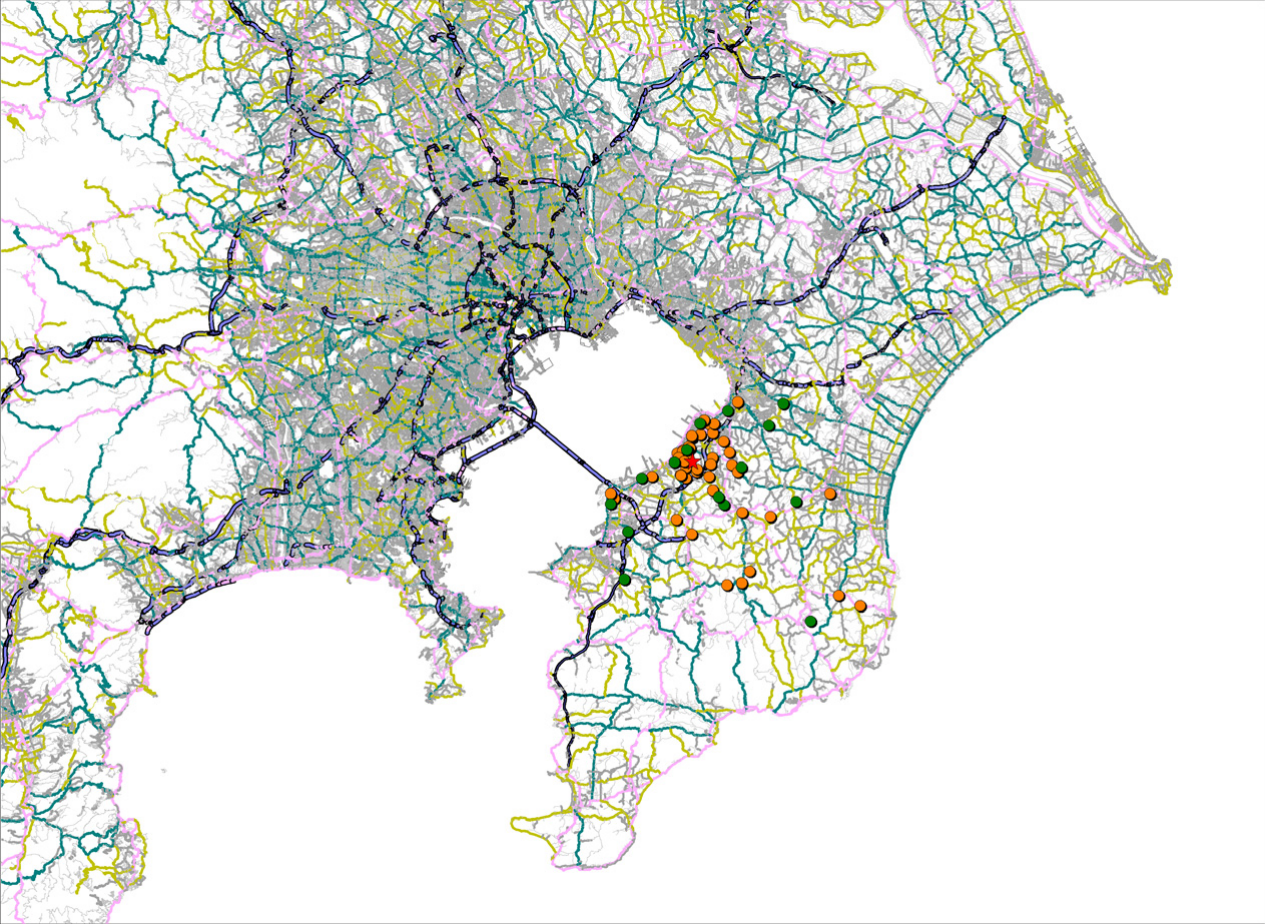
Address points of stomach (orange), liver (green) cancer patients, and the hospital (red).

**Figure 2:**
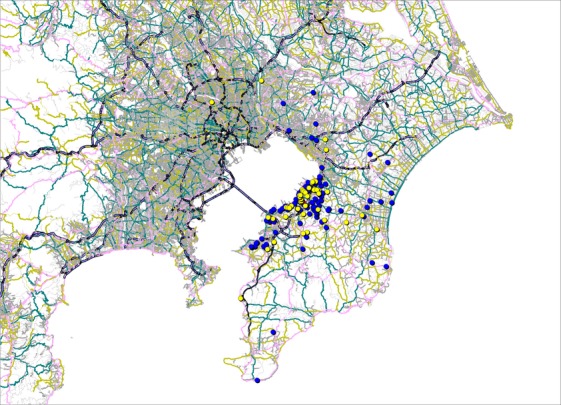
Address points of lung (yellow), colon (blue) cancer patients, and the hospital (red).

**Figure 3:**
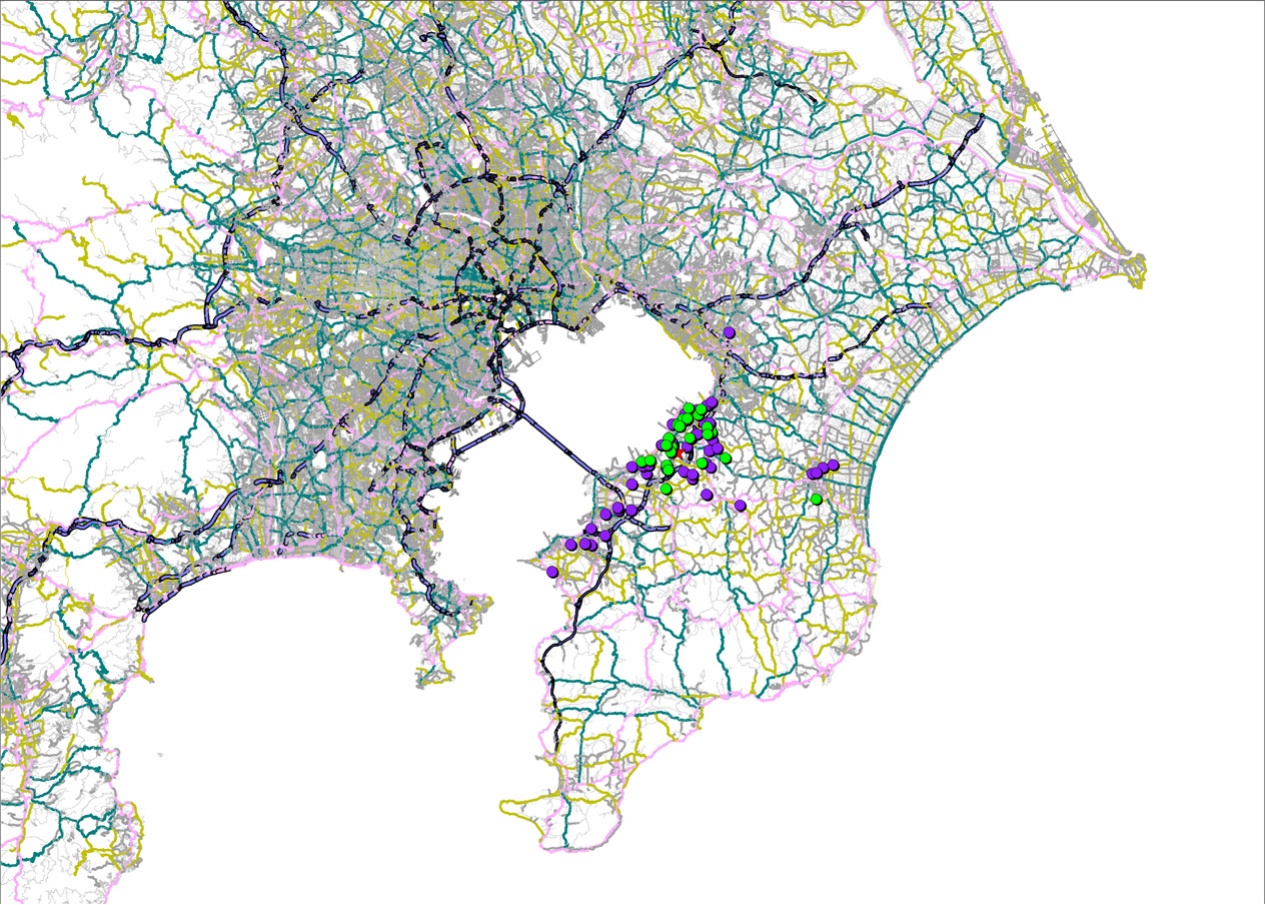
Address points of pancreas (light green), uterine (purple) cancer patients, and the hospital (red).

**Figure 4:**
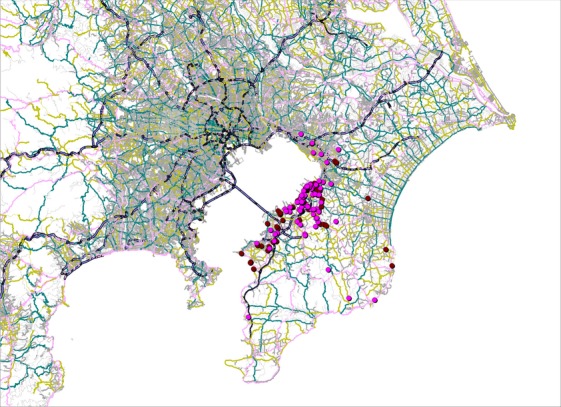
Address points of breast (pink), prostate (brown) cancer patients, and the hospital (red).

**Figure 5:**
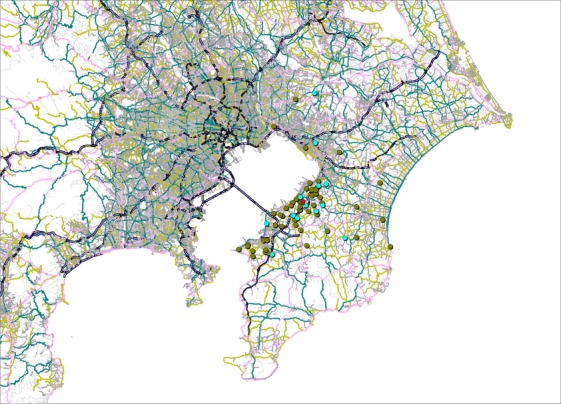
Address points of malignant lymphoma (olive), multiple myeloma (light blue) patients, and the hospital (red).

The maps display the address points of patients by cancer type (Figs. 1–5). Average round-trip travel distances, times, and costs by cancer type are shown in the Table 1. Average round-trip travel distance, time, and cost for all patients were 26.7 km, 72.5 min, and $79.99, respectively.

**Table 1:** Average round-trip travel distances, times, and costs by cancer type.

Cancer site	Stomach (*n* = 47)	Liver (*n* = 15)	Pancreas (*n* = 25)	Uterus (*n* = 55)	Breast (*n* = 86)	Prostate (*n* = 51)	Colon (*n* = 123)	Lung (*n* = 49)	Malignant lymphoma (*n* = 55)	Multiple myeloma (*n* = 15)
Age (range/median)	68.8 (39–87/69)	66.7 (56–81/66)	67.1 (39–83/68)	57.2 (24–80/59)	57.3 (33–84/57)	71.6 (39–89/72)	63.3 (33–83/63)	66.7 (36–82/70)	61.0 (5–84/65)	66.9 (48–81/63)
Distance (km) (range/median)	19.4 (2.2–82.0/12)	31.4 (4.2–75.2/25.2)	1.4 (2.0–60.2/13.0)	24.6 (2.4–71.4/16.8)	22.5 (0.8–117.6/15.9)	31.6 (0–94.2/24.0)	29.5 (0.4–165.4/18.8)	27.1 (1.0–142.0/17.6)	33.7 (1.4–99.6/23.8)	28.6 (0.4–98.6/18.8)
Time (min) (range/median)	54.7 (10–160/42)	87.1 (18–154/82)	47.6 (8–140/46)	70.0 (10–166/56)	63.2 (4–238/52)	83.4 (0–182/66)	77.3 (2–296/62)	74.3 (4–342/60)	88.1 (6–230/72)	77.9 (2–232/62)
Cost ($) (range/median)	58.1 (6.6–246.1/36.0)	94.4 (12.6–225.7/75.6)	43.3 (6.0–180.7/39.0)	73.9 (7.2–214.3/50.4)	67.6 (2.4–352.9/47.7)	94.7 (0–282.7/72.0)	88.7 (1.2–496.4/56.4)	81.2 (3.0–426.2/52.8)	101.2 (4.2–298.9/71.4)	85.7 (1.2–295.9/56.4)

## Discussion

Moore reported that out-of-pocket costs (including transportation fee, over-the-counter medications, and insurance premiums) about $360 per month [3]. We estimated that travel cost, which accounts for a significant part of out-of-pocket costs, is $40–$100.

The Medicare Prescription Drug, Improvement, and Modernization Act of 2003 (MMA) altered the guidelines for reimbursements for outpatient chemotherapy drugs and drug administration services in the United States. Friedman et al. reported that access to care between pre-MMA and post-MMA patients has no statistically significant difference [4]. Shea et al. also reported that median travel distance for chemotherapy is 11.2–12.8 km. Travel distance for chemotherapy in the Medicare population has not seen major changes after the enactment of the MMA [5].

## Limitation

As this study was performed only in a single facility, a meaningful tendency in travel time and costs was not seen in relation to the number of patients and specialists for each cancer type. A study in multiple facilities may show a notable trend.

## Conclusions

Cancer patients incurred a travel time of 45–90 min and a cost of $40–$100 for each outpatient treatment. Cancer patients carry rising burdens of health care-related out-of-pocket expenses. With population aging, seniors living alone, and increasing number of senior households, outpatient visits are becoming a common burden.
